# Frequent unregulated use of antibiotics in rural Cambodian infants

**DOI:** 10.1093/trstmh/traa020

**Published:** 2020-04-20

**Authors:** Asuka Miyazaki, Rathavy Tung, Bunsreng Taing, Mitsuaki Matsui, Azusa Iwamoto, Sharon E Cox

**Affiliations:** 1 School of Tropical Medicine and Global Health, Nagasaki University, Nagasaki, Japan; 2 National Maternal and Child Health Centre, Ministry of Health, Phnom Penh, Cambodia; 3 Kampong Cham Provincial Health Department, Cambodia; 4 Bureau of International Health Cooperation, National Center for Global Health and Medicine, Tokyo, Japan; 5 Institute of Tropical Medicine, Nagasaki University, Nagasaki, Japan; 6 Dept of Population Health, London School of Hygiene and Tropical Medicine, London, UK

**Keywords:** Cambodia, antibiotics, infants

## Abstract

**Background:**

Despite a high prevalence of antibiotic resistance in Cambodia, few studies have assessed health-seeking behaviour and the use of antibiotics by caregivers of young children in Cambodia.

**Methods:**

We conducted a cross-sectional survey of infants <12 months of age and their caregivers, assessing the frequency of reported illness, common symptoms and associated health-seeking behaviour through structured questionnaires administered by trained fieldworkers at a home visit. In a subset of these participants, ages 4–8 months with no acute malnutrition, we conducted a 3-month surveillance with fortnightly home visits.

**Results:**

Of 149 infants (ages 1–11 months, 54.4% male) enrolled in the cross-sectional study, 76 (51.4%) reported symptoms of diarrhoea, fever or cough in the previous 14 d, with associated use of antibiotics reported in 22 (14.8%) infants. In 47 infants enrolled in the longitudinal surveillance, there were 141 reported episodes of illness in 44 (94%) infants with 21 infants (45%) reported to have received antibiotics in 32/141 (22.7%) episodes. Amoxicillin was the most commonly reported antibiotic in both surveys (68% [40/59 episodes reporting the use of antibiotics]).

**Conclusions:**

Antibiotic usage is high in this population and appears to be occurring largely outside of the formal healthcare system.

## Introduction

Inappropriate antibiotic use can lead to the development of antimicrobial resistance (AMR). This is a global problem, but one likely to have the most impact on the poorest populations where infectious diseases are still highly endemic. Data on the prevalence of AMR in Cambodia is still limited but suggests a potentially high prevalence in common pathogenic organisms to important antibiotics, including ampicillin, third-generation cephalosporins and fluroquinolones.^1^ The western border area of Cambodia is known to be a hotspot for the development of new resistance-causing mutations for antimalarial drugs^2^ and there is increasing research on health provider and community use of antimalarials,^3^ but much less for antibiotics, particularly within the community. Inappropriate prescribing of antibiotics by healthcare professionals in Cambodian hospitals has been documented and perceived patient demand is cited as one of the reasons for high rates of prescription.^4^ Exposure to antibiotics in infancy can alter the establishment of the intestinal microbiota,^5^^,^^6^ while antibiotic use has been associated with a range of long-term adverse health outcomes.^7^

During a cross-sectional study and 3-month surveillance of a subgroup we aimed to describe the incidence of episodes of diarrhoea, fever and cough as reported by mothers and describe associated health-seeking behaviour and the proportion of episodes associated with reported antibiotic use or other medications.

## Methods

### Study area, design and populations

The study area comprised 12 small villages along the Mekong River in Stueng Trang district, Kampong Cham province, which is about 60 km from the provincial capital, with limited transport (Supplemental Figure 1A–C). Residents in the area are mostly small-scale farmers. The area is not a known malaria-affected area. The current exploratory research was conducted within the context of an ongoing cohort study known as the Nutrition for Health of *Aka-chan* (baby in Japanese) and Mamas (hereafter referred to as the NHAM birth cohort), which enrolled all singleton newborn infants born between April 2016 and March 2019, following them until they are 2 y old, to investigate causes of chronic malnutrition.

A cross-sectional survey was conducted in children previously enrolled in the NHAM birth cohort. From within the survey population we invited caregivers of children ages 4–<9 months with no acute malnutrition (weight-for-length z-score (WLZ) >−2 and no bilateral pitting oedema) and who planned to stay resident within the study area for the duration of the study to participate in a 3-month detailed longitudinal follow-up study focussing on the effects of weaning and hygiene practices on growth and infections. This report is limited to the results concerning the number of reported episodes of illness and associated health-seeking behaviour.

### Data collection

After pilot testing and revision of the questionnaires (in English and Khmer), trained fieldworkers with a certificate in either nursing, midwifery or medicine administered paper-based structured questionnaires, translated into the local language (Khmer), to primary caregivers of enrolled infants during home visits and conducted anthropometric measurements. In the longitudinal study, the same fieldworkers visited the primary caregiver in their homes every 2 weeks and administered a structured questionnaire and conducted anthropometric measurements at the first and last visit. Caregivers were asked if the enrolled child had been unwell in the previous 14 d (cross-sectional survey) or since the previous visit, or in the case of the first follow-up visit, in the previous 7 d. If the response was ‘no’, no further questions were asked. If the caregiver reported that the child had been unwell they were questioned about the occurrence and duration of diarrhoea (more than three loose stools in 24 h), cough and fever. Caregivers were then asked questions about if and where medical advice was sought and what medications were given by asking first if the child had taken any intervention from any source for each episode of illness and fieldworkers selecting the closest response from the options provided. If the caregiver responded that the child had received an intervention, they were asked what kind, and based on the caregivers response and probing by the fieldworker, the closest response from the options provided was selected, including ‘other’ and details (please see the supplementary material). Where possible, fieldworkers directly observed drug packaging or obtained pictures of the drug to confirm the name of the drug. Separate episodes of illness were defined as those occurring with at least 1 d apart with no symptoms.

### Data management and analysis

Data were entered into EpiData Entry Client (version 1.4.2, EpiData Association, Odense, Denmark) and descriptive analysis was conducted using Stata 14.2 (StataCorp, College Station, TX, USA). No formal sample size calculation was performed for this exploratory work, as the sample size was determined by the parent NHAM cohort.

### Ethics statement

All participants gave written informed consent for participation in each of the studies. Before giving consent, trained fieldworkers provided participants with information about the study in the local language (Khmer) and participants were given the opportunity to ask questions. Fieldworkers explained that participants had the right to withdraw from the study at any time without giving a reason and that non-participation would not affect the clinical care of anyone in their household. This study was approved by the Ethics Committee of the Nagasaki University School of Tropical Medicine and Global Health (NU-TMGH-007) and the National Ethical Committee for Health Research (NECHR) in the Ministry of Health, Cambodia (no. 135). The NHAM birth cohort study was approved by both the NECHR in the Ministry of Health, Cambodia (no. 016) and the Ethical Committee of the National Centre for Global Health and Medicine in Japan (NCGM-G-001870-01).

## Results

**Figure 1:**
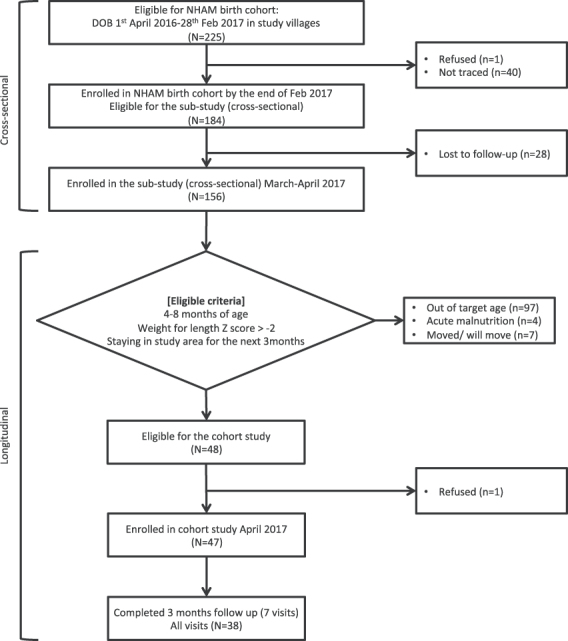
Flow chart of study participants.

A total of 156 children from the NHAM cohort were enrolled in the cross-sectional survey between March and April 2017 (Figure 1). Of these, 149 had complete and consistent data on recent illness and were included in this analysis, with a mean age of 5.6 months (standard deviation [SD] 3.0; range 1.1–11.9) and included 80 males (53.7%). Of 48 infants eligible for the longitudinal surveillance study, forty-seven infants were enrolled in April 2017, with a mean age of 6.5 months (SD 1.1; range 4.7–8.6) and included 25 (53%) males. The median duration of follow-up was 96 d [interquartile range 90–103; minimum/maximum: 20/111], with 38/47 (81%) children traced at all seven planned follow-up visits and 42 children completing the final follow-up visit (Figure 1).

The number of children and episodes with different symptoms and reported use of antibiotics and probiotics are shown in Table 1. In the cross-sectional survey, 81 children were reported to have been unwell in the previous 14 d, 76 with symptoms of diarrhoea, fever or cough (Table 1). In the longitudinal surveillance there were 141 episodes of illness including symptoms of diarrhoea, fever, cough or other symptoms documented during 305 home-visits (Table 1). Only three children (6%) did not experience any episodes. In the cross-sectional survey, the most common episodes were those with fever and cough, accounting for 34.6% (28/81) of all illness episodes. In the longitudinal surveillance, fever alone was the most common episode (31.2% [44/141]). The use of antibiotics was common, occurring in 14.8% (22/149) of children in the cross-sectional study and 45% (21/47) of children and 22.7% (32/141) of all episodes in the longitudinal surveillance (Table 1). Of the 21 children with reported antibiotic use during the longitudinal surveillance, 7 children had two episodes and two children had three episodes with reported antibiotic use. In both studies, the use of antibiotics in episodes with diarrhoeal symptoms was more frequent than the use of zinc and/or oral rehydration solution (ORS), which was reported in 2/17 (12%) and 5/38 (13%) episodes compared with antibiotic use in 4/17 (24%) and 7/38 (18%) of diarrhoeal episodes in the cross-sectional and longitudinal surveillance studies, respectively. There were also seven instances of reported use of commercially available probiotic products, all in diarrhoeal episodes during the longitudinal surveillance, but none in the cross-sectional study.

**Table 1:** Frequency of illness episodes and reported use of antibiotics

Type of episode		Cross-sectional survey (149 children)^a^			Longitudinal surveillance (305 visits in 47 children)^b^		
		Children with illness episodes, n (%)	Episodes with antibiotics, n (%)	Episodes with probiotic, n (%)	Visits with illness episodes, n (%)	Episodes with antibiotics, n (%)	Episodes with probiotics, n (%)
Each episode with or without other symptom	Diarrhoea	17 (11.4)	4 (23.5)	0	38 (12.5)	7 (18.4)	7 (18.4)
	Cough	51 (34.2)	15 (29.4)	0	71 (23.3)	18 (25.4)	2 (2.8)
	Fever	53 (35.6)	18 (34.0)	0	102 (33.4)	27 (26.5)	3 (2.9)
Symptom combinations during episodes of illness	Diarrhoea only	6 (4.0)	0	0	12 (3.9)	0	4 (25.0)
	Cough only	14 (9.4)	3 (21.4)	0	20 (6.6)	4 (20.0)	0
	Fever only	17 (11.4)	6 (35.3)	0	44 (14.4)	9 (20.5)	0
	Fever and cough	28 (18.8)	9 (32.1)^c^	0	35 (11.5)	11 (31.4)^c^	0
	Fever and diarrhoea	2 (1.3)	1 (50.0)	0	10 (3.3)	4 (40.0)^d^	1 (10.0)
	Diarrhoea and cough	3 (2.0)	1 (33.3)	0	3 (1.0)	0	0
	Diarrhoea, fever and cough	6 (4.0)	2 (33.3)	0	13 (4.3)	3 (23.1)	2 (15.4)
	Other	5 (3.4)^e^	0	0	4 (1.3)^f^	1 (25.0)	0
	Total	81/149 (54.4)	22/81 (27.2)	0/81	141 (46.2)	32/141 (22.7)	7/141 (5.1)

^a^Reported illness episode during the previous 14 d.

^b^Reported illness episodes occurring during 3-month surveillance with fortnightly home visits.

^c^Two episodes had two different reported antibiotics.

^d^One episode had two different reported antibiotics.

^e^Four episodes with runny nose and one episode with ear infection.

^f^Two episodes with runny nose, one episode with hypothermia and one episode with oral infection.

The frequency of use for each antibiotic for the two studies combined is shown in Table 2. The most common antibiotic used was amoxicillin in 19/24 instances in the cross-sectional study and 21/35 instances in the longitudinal surveillance and was most commonly associated with the presence of fever.

**Table 2:** Frequency of use of different reported antibiotics by the presence of symptoms in 54 episodes of illness

Antibiotic group		Medicine	Frequency, n (%) (N=59)^a^	No instances used for each symptom occurrence			
				Fever	Cough	Diarrhoea	Others
Penicillin		Amoxicillin	40 (67.8)	35	23	5	1
Cephems	First generation	Cefadroxil	6 (10.2)	4	6	1	0
	Second generation	Cefaclor	3 (5.1)	1	3	1	0
	Third generation	Cefixime	3 (5.1)	3	0	2	0
		Ceftriaxone	1 (1.7)	1	1	0	0
Macrolides		Erythromycin	1 (1.7)	1	1	0	0
New quinolones		Ofloxacin	1 (1.7)	1	0	1	0
Tetracyclines		Tetracycline	2 (3.4)	2	2	0	0
Others		Co-trimoxazole	1 (1.7)	1	1	1	0
		Metronidazole	1 (1.7)	1	0	1	0

^a^Five episodes received two antibiotics simultaneously: metronidazole and ofloxacin (one episode with fever and diarrhoea), ceftriaxone and amoxicillin (one episode with fever and cough), cefadroxil and amoxicillin (one episode with fever and cough) and tetracycline and amoxicillin (two episodes with fever and cough).

Health-seeking behaviour is described in Table 3. Home treatment or no health-seeking behaviour was reported in 11.1% (9/81) and 20.6% (29/141) of episodes in the cross-sectional survey and longitudinal surveillance, respectively. However, three home-treated episodes also reported antibiotic use, implying that these were already available in the home at the time of the illness episode. The most common health-seeking behaviour during an episode of illness was to seek advice or treatment from the private residence of a nurse or midwife residing in the local area, including during episodes of reported antibiotic use.

**Table 3:** Frequency and types of health-seeking behaviour

	149 children at baseline survey		47 children during surveillance	
Health-seeking behaviour	All episodes, n (%) (N=81)	Episodes with reported antibiotic use, n (%) (N=22)	All episodes, n (%) (N=141)	Episodes with reported antibiotic use, n (%) (N=32)
Sought advice/treatment from outside	72 (88.9)	22 (100)	112 (79.4)	29 (90.6)
None or treatment from home	9 (11.1)	0	29 (20.6)	3 (9.4)
Episodes with advice/treatment outside of home	(N=72)	(N=22)	(N=112)	(N=29)
Public facility				
Health centre	25 (29.4)	10 (37.0)	22 (17.7)	6 (18.2)
Hospital	1 (1.2)	1 (3.7)	4 (3.2)	1 (3.0)
Private facility				
Grocery store	5 (5.9)	0	3 (2.4)	1 (3.0)
Pharmacy or drug shop	8 (9.4)	0	11 (8.9)	3 (9.1)
Nurse/midwife residence	41 (48.2)	14 (51.9)	72 (58.1)	17 (51.5)
Clinic	5 (5.9)	2 (7.4)	11 (8.9)	4 (12.1)
Hospital	0	0	1 (0.8)	1 (3.0)
Total health-seeking behaviour events^a^	85	27	124	33

^a^Some caregivers visited more than one place to seek advice/treatment.

## Discussion

We demonstrate a high incidence of symptoms of illness in young rural Cambodian infants resulting in health-seeking behaviour outside of the home and frequent use of antibiotics. During 3 months of surveillance of infants 3–9 months of age, slightly less than half had reportedly received one or more antibiotics, most of which were apparently obtained outside of the formal health system. A previous study of paediatric outpatients at a tertiary hospital in Cambodia in 2011 reported that 37% of 775 children (mean age 4.6 y) had received antibiotics prior to presentation as detected by urinary antibacterial activity, compared with only 7.3% by caregiver report, although 69% of caregivers reported giving one or medications prior to attendance at the outpatient department. Similar to our study, the most common antibiotic was amoxicillin, with the most common reported source being private pharmacies, and antibiotic use was most often associated with reported fever.^8^ Data from the 2014 Cambodian Demographic and Health Survey reported that 38% of children ages 6–11 months had reported fever in the previous 2 weeks and that 82% of these were reported to have taken antibiotic treatment.^9^

Detailed follow-up of morbidity and reported antibiotic use (validated against medical records in a subset) was conducted within the multicountry Malnutrition and Enteric Disease Study (MAL-ED) birth cohort, conducted in eight countries, including Bangladesh, Nepal, Pakistan and India in Asia, with very high antibiotic use reported in Pakistan at 11.9 courses per child year compared with the study mean of 4.9 courses per child year. In all sites, antibiotic usage was highest at 6–11 months of age. In Pakistan, 50% of children had received one or more antibiotic courses before 1 month of age.^10^ However, in this study the source of antibiotics was not reported, but non-prescription antibiotic use is known to be common in many low-income countries, especially in Asia,^11^ including in Cambodia.^9^

**Figure 2:**
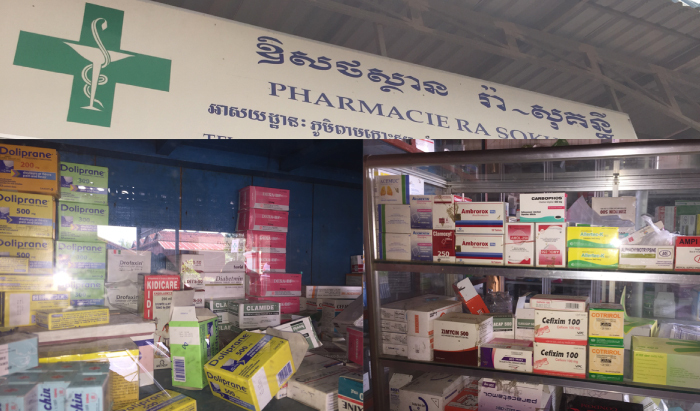
Medicines available at a local drug shop.

Despite our study area being rural with few shops, we documented during this research the presence of several drug shops (Figure 2). However, in our study population the most common place where caregivers obtained antibiotics was the private residence of nurses/midwives in the community, which is a common health-seeking behaviour in rural Cambodia.^4^^,^^9^ Residing in these villages were a mix of government nurses/midwives and some who worked entirely privately. When caregivers reported obtaining drugs from the private residence of a nurse/midwife, we are confident that this is distinct from visiting the government clinic at which the nurse might be working, but what we could not determine was the source and documentation of the antibiotics supplied in this way.

In our study population, the symptom most frequently associated with the use of antibiotics was fever, followed by cough, similar to results previously reported in Cambodia.^8^ The frequency of use of zinc and ORS in episodes of reported diarrhoea was very low and less frequent than the use of antibiotics or commercially available probiotic drinks, which were used in some cases of diarrhoea (but not in the absence of diarrhoea).

**Figure 3:**
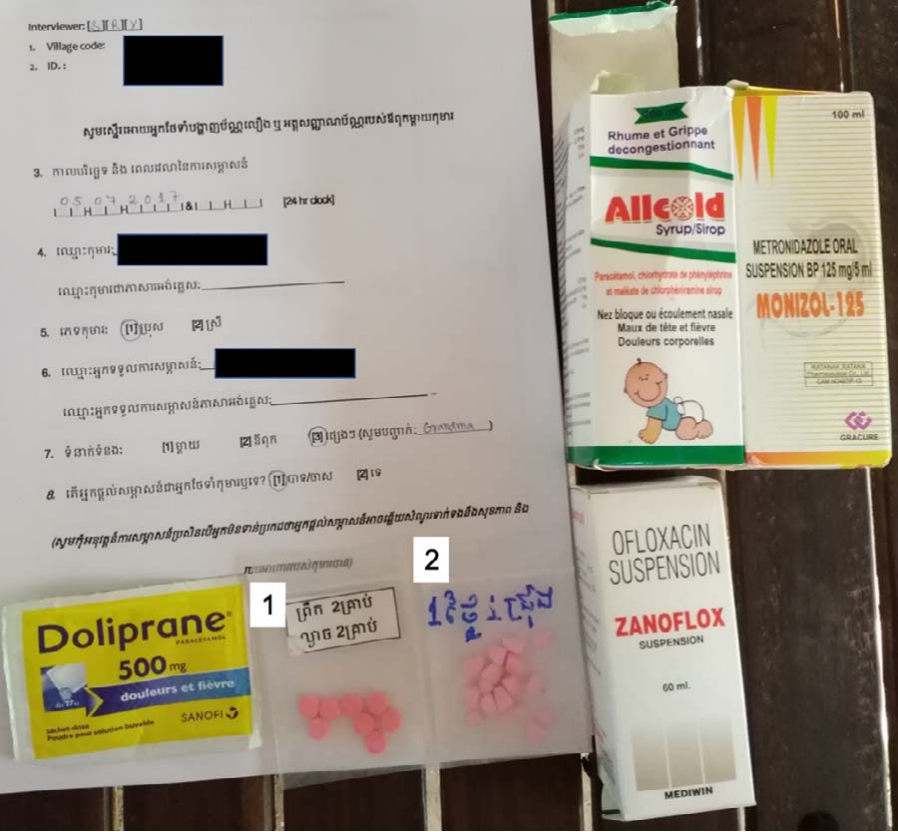
Medicines taken during a single episode of illness. 1: ‘Take 2 pills morning and evening’; 2: ‘Take ¼ pill per day’.

Our observations are in line with those from a qualitative study in Cambodia seeking to explore adult antibiotic-seeking behaviour in the community and the drivers of misuse.^4^ The authors reported the common use of antibiotics for mild illnesses, community familiarity with antibiotics such that the names of many were used in conversation, frequent self-prescription and common provision of antibiotics through nurses from public facilities offering private, out-of-hours services. Misuse also commonly included the purchase of only one or two doses and of inappropriate cocktails. The authors concluded ‘misuse of antibiotics was widespread…driven by a determined antibiotic-seeking behaviour facilitated by trained and untrained healthcare providers who supplied and enabled the community’s antibiotic use’. Figure 3 shows an example of the medications, including antibiotics, that were given during one episode of fever and diarrhoea, showing the use of both formally and informally packaged medicines.

The limitations of the current study include the lack of a measure of the severity of illness episodes and of objective assessment of illness symptoms that would allow the appropriateness of antibiotic use to be assessed in relation to the Integrated Management of Childhood Illness guidelines,^12^ for example, bloody vs watery diarrhoea. We also sometimes had to rely on caregivers reports of what medications/antibiotics were given, as many were informally packaged in small bags (Figure 2), sometimes without handwritten labels or instructions. Nor was information collected on the number and dosage of the antibiotics that were obtained and then given, which prevented us from calculating the number of days exposed to antibiotics per child observation days. Given the relatively small number of children and observations and the small sample size for this exploratory analysis, we did not attempt any formal statistical testing of associations. The lack of validation of caregiver-reported antibiotic use could have resulted in an over- or underestimation of the true community use of antibiotics in this population. However, in this setting it was apparent that there are few medical records against which to validate self-reports, while analysis of urinary antibiotic activity will only pick up antibiotic consumption in the previous 1–3 d and would require many more home visits and be burdensome for the study participants to complete. It is possible that those who had more missed visits or who could not be traced for participation could have resulted in selection bias.

Further studies using mixed methods are required to document and understand antibiotic health-seeking behaviour for caregivers of young children in Cambodia and to investigate the impacts of these behaviours on AMR, gut health and the microbiome and in preventing children from receiving more appropriate community level care such as ORS and zinc for diarrhoeal episodes.

## Supplementary Material

traa020_Supplemental_Figure

## References

[1] Reed TAN, Krang S, Miliya T et al. Antimicrobial resistance in Cambodia: a review. Int J Infect Dis. 2019;85:98–107. 31176035 10.1016/j.ijid.2019.05.036

[2] Nosten FH, Phyo AP New malaria maps. Lancet. 2019;394(10195):278–279. 31229232 10.1016/S0140-6736(19)31273-5

[3] Littrell M et al. Case management of malaria fever in Cambodia: results from national anti-malarial outlet and household surveys. Malar J. 2011;10:328. 22039922 10.1186/1475-2875-10-328 PMC3224783

[4] Om C et al. *“If it’s a broad spectrum, it can shoot better”*: inappropriate antibiotic prescribing in Cambodia. Antimicrob Resist Infect Control. 2016;5:58. 28031814 10.1186/s13756-016-0159-7 PMC5170903

[5] Korpela K, Salonen A, Saxen H et al. Antibiotics in early life associate with specific gut microbiota signatures in a prospective longitudinal infant cohort. Pediatr Res. 2020. doi: 10.1038/s41390-020-0761-5. 31954376

[6] Bokulich NA et al. Antibiotics, birth mode, and diet shape microbiome maturation during early life. Sci Transl Med. 2016;8(343):1–14. 10.1126/scitranslmed.aad7121 PMC5308924 27306664

[7] Valdes AM et al. Role of the gut microbiota in nutrition and health. BMJ. 2018;361:k2179. 29899036 10.1136/bmj.k2179 PMC6000740

[8] Emary KR et al. Urinary antibiotic activity in paediatric patients attending an outpatient department in north-western Cambodia. Trop Med Int Health. 2015;20(1):24–28. 25324202 10.1111/tmi.12398 PMC4284023

[9] National Institute of Statistics, Directorate General for Health, ICF International. Cambodia Demographic and Health Survey 2014. Phnom Penh, Cambodia and Rockville, MD, USA: National Institute of Statistics, Directorate General for Health and ICF International; 2015 Available at: https://dhsprogram.com/pubs/pdf/FR312/FR312.pdf.

[10] Rogawski ET et al. Use of antibiotics in children younger than two years in eight countries: a prospective cohort study. Bull World Health Org. 2017;95(1):49–61. 28053364 10.2471/BLT.16.176123 PMC5180352

[11] Morgan DJ et al. Non-prescription antimicrobial use worldwide: a systematic review. Lancet Infect Dis. 2011;11(9):692–701. 21659004 10.1016/S1473-3099(11)70054-8 PMC3543997

[12] World Health Organization. Handbook: IMCI integrated management of childhood illness. Geneva World Health Organization, 2005 Available at: https://www.who.int/maternal_child_adolescent/documents/9241546441/en/

